# Elucidating the novel BRCA1 function as a non-genomic metabolic restraint in ER-positive breast cancer cell lines

**DOI:** 10.18632/oncotarget.26093

**Published:** 2018-09-11

**Authors:** Moses Koobotse, Jeff Holly, Claire Perks

**Affiliations:** ^1^ IGFs and Metabolic Endocrinology Group, Translational Health Sciences, University of Bristol, Bristol, UK; ^2^ Faculty of Health Sciences, School of Allied Health Professions, University of Botswana, Gaborone, Botswana

**Keywords:** BRCA1, IGF-I, cancer, p-ACCA, non-genomic

## Abstract

Within populations carrying the same genetic predisposition, the penetrance of *BRCA1* mutations has increased over time. Although linked to changes in lifestyle factors associated with energy metabolism, these observations cannot be explained by the established role of BRCA1 in DNA repair alone.

We manipulated BRCA1 expression using tetracycline in the UBR60-bcl2 cell line (which has an inducible, tetracycline-regulated BRCA1 expression) and siRNA in oestrogen receptor(ER)-positive MCF7 and T47D breast cancer cells. Cellular responses to BRCA1 silencing and IGF-I actions were investigated using western blotting, 3-H Thymidine incorporation assay, cell fractionation and co-immunoprecipitation.

We demonstrated that the loss of BRCA1 resulted in downregulation of a phosphorylated and inactive form of acetyl CoA Carboxylase-α (ACCA), with a concomitant increase in fatty acid synthase (FASN) abundance. BRCA1 was predominantly cytoplasmic in ER-positive breast cancer cells, compatible with the observation that BRCA1 physically associates with phosphorylated ACCA, which is a cytoplasmic protein. We also found that IGF-I induced de-phosphorylation of ACCA by reducing the interaction between BRCA1 and phosphorylated ACCA. BRCA1 deficiency enhanced the non-genomic effects of IGF-I, as well as the proliferative responses of cells to IGF-I.

We characterized a novel, non-genomic role for BRCA1 in restraining metabolic activity and IGF-I anabolic actions.

## INTRODUCTION

The breast cancer 1, early onset (*BRCA1)* gene is frequently mutated in familial breast cancers and women harbouring these germline mutations have an increased risk of developing breast cancer [1, 2]. Although somatic mutations are rare in sporadic breast cancers, BRCA1 dysfunction has also been reported in a large number of these cases [3–5]. The *BRCA1 gene* product has established roles in the maintainance of genome integrity [6–8], however, the increasing penetrance of *BRCA1* mutations over time within populations carrying the same genetic predisposition [2, 9] suggests that BRCA1 may be involved in other roles beyond the genome. Indeed, BRCA1 has recently been shown to play a regulatory role in lipogenesis by interacting with Acetyl CoA carboxylase-α (ACCA) in a phospho-dependent manner [10–12].

Acetyl CoA carboxylase exists as ACCA and acetyl CoA carboxylase-ß (ACCB) isoforms, with ACCA involved in *de novo* fatty acid synthesis in the cytosol whereas ACCB regulates mitochondrial fatty acid oxidation on the outer mitochondrial membrane [13]. In the fatty acid synthesis pathway, ACCA catalyses the rate-limiting step of the pathway and generates substrates for fatty acid synthase (FASN) [14]. Phosphorylation of a number of serine residues has been shown to regulate ACCA by reducing its activity [15, 16] and among these, Ser-79 has been identified as a major regulatory site [17]. The formation of a complex between BRCA1 and p-ACCA (S^79^) prevents dephosphorylation of ACCA [10], thus acting as a brake on tumour cell anabolism and proliferation. Disruption of this complex due to BRCA1 protein dysfunction resulting from either *BRCA1* gene mutation or epigenetic silencing, may have huge implications for tumour cell anabolism.

Insulin-like growth factor-I (IGF-I) is a peptide hormone that plays an important role in normal mammary growth and development and its effects are mainly mediated through the type 1 IGF receptor (IGF-IR) [18]. In many epithelial tumours, including those of the breast, high circulating levels of IGF-I have been correlated with increased cancer risk and the IGF-IR is frequently overexpressed in the tumours [19, 20]. These observations suggest that IGF-I and its receptor play a key role in carcinogenesis. Additionally, the IGF-I/IGF-IR signalling pathway negatively correlates with BRCA1 abundance in breast and prostate cancer cells [21, 22], suggesting an interplay between BRCA1 and IGF-I/IGF-IR signalling. We recently made a novel observation that IGF-I regulates FASN abundance in non-malignant mammary epithelial cells and estrogen receptor (ER)-positive breast cancer cells [23]. However, the proliferative response to IGF-I was only dependent upon FASN in ER-positive breast cancer cells and not in non-malignant breast epithelial cells. These data provided a link between an established role of IGF-I as a cell cycle regulator with a role in lipogenesis. Although BRCA1 may also regulate cell cycle through its role in lipogenesis [15], little is known about the extent of BRCA1 involvement in the fatty acid synthesis pathway and how BRCA1 deficiency may impact on IGF-I actions.

In this study, we report that BRCA1 is predominantly localised to the cytoplasm in ER+ breast cancer cells where it associates with p-ACCA (S^79^). In cells expressing full-length, wildtype BRCA1, IGF-I induces dephosphorylation of ACCA by reducing the interaction between BRCA1 and p-ACCA (S^79^), with a concomitant increase in FASN abundance downstream of ACCA. BRCA1 deficiency also results in a reduction in the inactive form of ACCA and subsequently, an increase in FASN. Additionally, BRCA1 deficiency enhances the non-genomic effects of IGF-I and the proliferative responses of cells to IGF-I. With the data presented here, we characterized a novel, non-genomic role for BRCA1 in breast tumour suppression, contributing to a growing list of emerging BRCA1 functions.

## RESULTS

### BRCA1 negatively regulates ACCA and FASN in UBR60-bcl2 and ER-positive breast cancer cells

**Figure 1:**
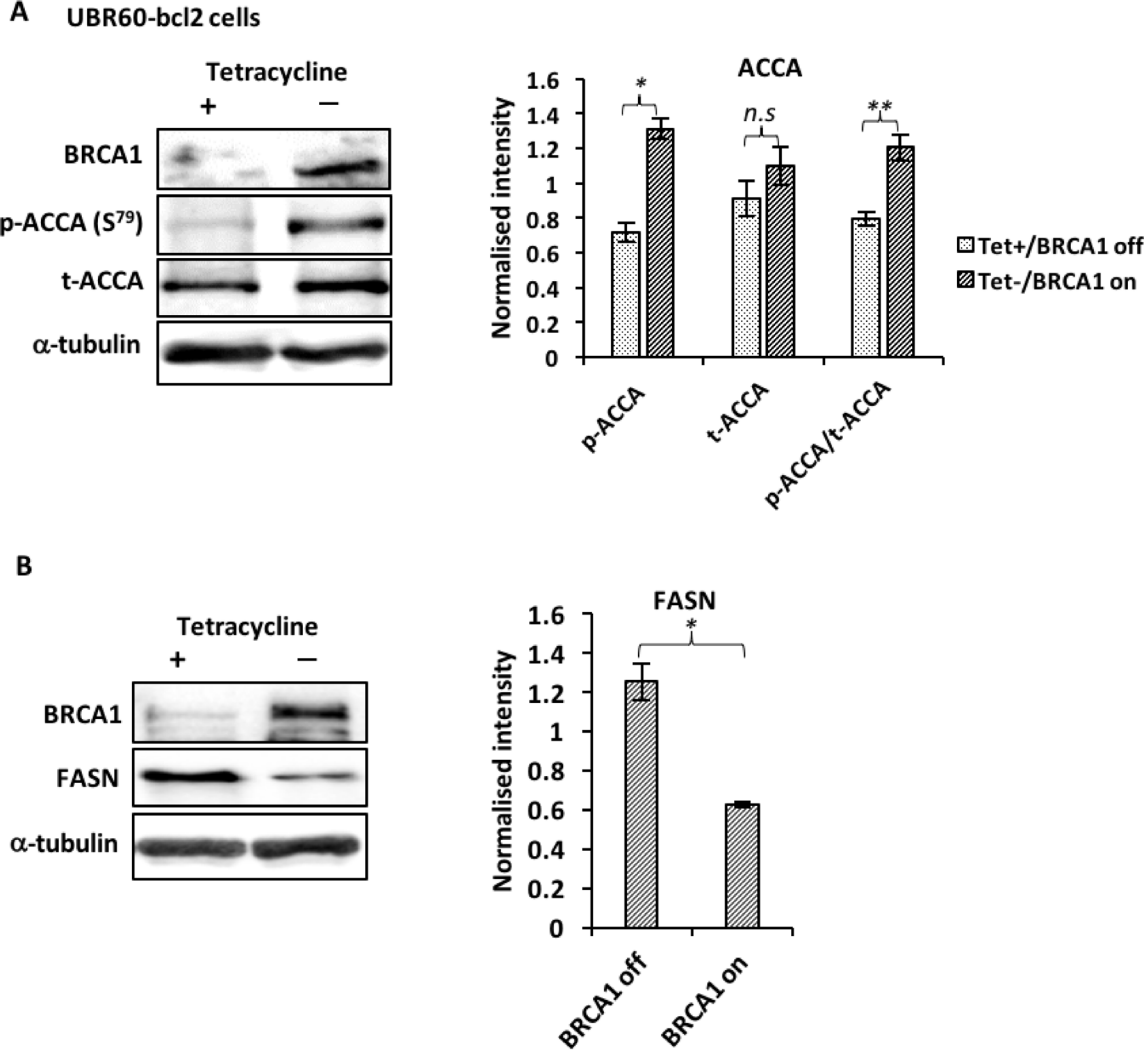
BRCA1 regulates ACCA and FASN in UBR60-bcl2 cells Cells cultured with or without tetracycline for 48 hours were lysed and analyzed by western blotting. (**A**) Representative immunoblots of BRCA1, p-ACCA (S^79^) and total ACCA from three independent experiments are shown. The graph shows densitometry analysis of total ACCA normalised to α-tubulin and p-ACCA (S^79^) normalised to both α-tubulin and total ACCA. (**B**) Representative immunoblots of BRCA1, FASN and α-tubulin from three independent experiments are shown. The graph shows densitometry analysis of FASN normalized to α-tubulin. All the densitometry data are expressed are expressed as mean ± S.E.M of three independent experiments. ^*^P < 0.05, ^**^P < 0.01 and ^***^p < 0.001, Student’s *t*-test (tet− versus tet+).

**Figure 2:**
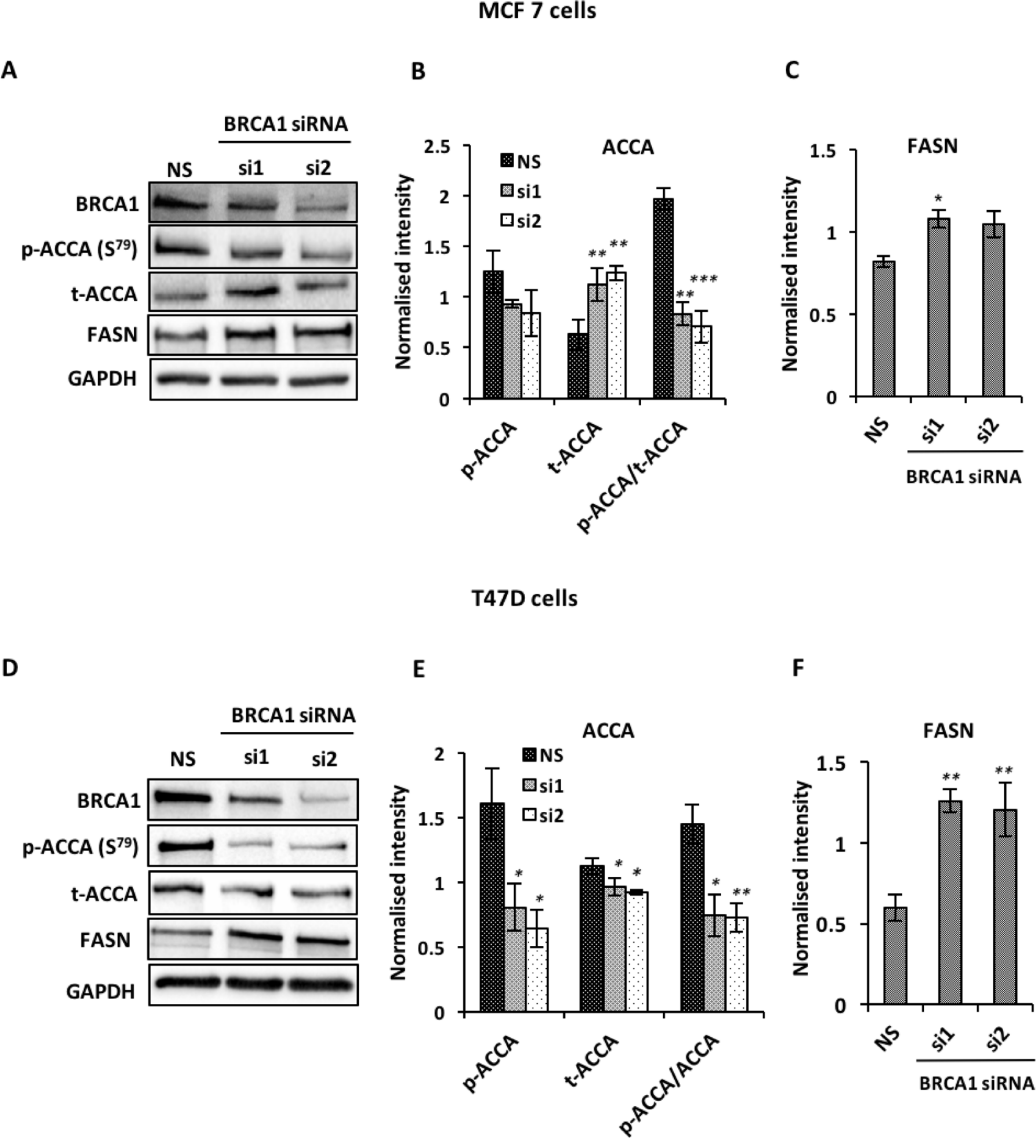
BRCA1 silencing up-regulates ACCA and FASN in MCF7 and T47D breast cancer cells Cells were transfected with two BRCA1 siRNAs (siRNA1 and siRNA2) targeting different sequences of BRCA1 or with non-silencing siRNA (NS) as negative control for 48 hrs. The abundance of BRCA1, p-ACCA (S^79^), total ACCA and FASN were analyzed using western blotting with GAPDH as a loading control. Representative blots are shown for (**A**) MCF7 and (**D**) T47D cells. (**B**, **E**) The graphs show densitometry analysis of total ACCA normalized to GAPDH and p-ACCA (S^79^) normalized to both GAPDH and total ACCA. (**C**, **F**) The graphs show densitometry analysis of FASN normalized to GAPDH. All the densitometry data are expressed are expressed as mean ± S.E.M of three independent experiments. ^*^P < 0.05, ^**^P < 0.01 and ^***^p < 0.001, one-way ANOVA.

Recent evidence showed that BRCA1 regulates lipogenesis through its interaction with the phosphorylated and inactive form of the ACCA enzyme [11, 24]. This interaction acts as a brake on lipogenesis by preventing dephosphorylation of ACCA, keeping it in this inactive state. We reasoned that BRCA1 abundance affects key enzymes of the fatty acid synthesis pathway. Using UBR60-bcl2 cell line with an inducible, tetracycline-regulated BRCA1 expression described previously [25], we show that BRCA1 induction resulted in an increase in ACCA phosphorylation (Figure 1A). In addition to increasing ACCA phosphorylation, the induction of BRCA1 also caused a significant reduction in FASN abundance (Figure 1B). These data suggest that BRCA1 favours the maintenance of ACCA in an inactive state and low FASN abudance downstream. To investigate whether the effects could be replicated in breast cancer cells, we used short interfering RNA (siRNA) to downregulate endogenous BRCA1 in ER-positive MCF7 and T47D breast cancer cell lines which have relatively high abundance of BRCA1 (Supplementary Figure 1). BRCA1 siRNA 1 and siRNA 2 targeting different sequences of the *BRCA1 gene*, reduced ACCA phosphorylation and increased FASN abundance in both MCF7 (Figure 2A–2C) and T47D (Figure 2D–2F) breast cancer cells.

### BRCA1 is predominantly cytoplasmic in ER-positive breast cancer cell lines and physically interacts with phosphorylated ACCA

**Figure 3:**
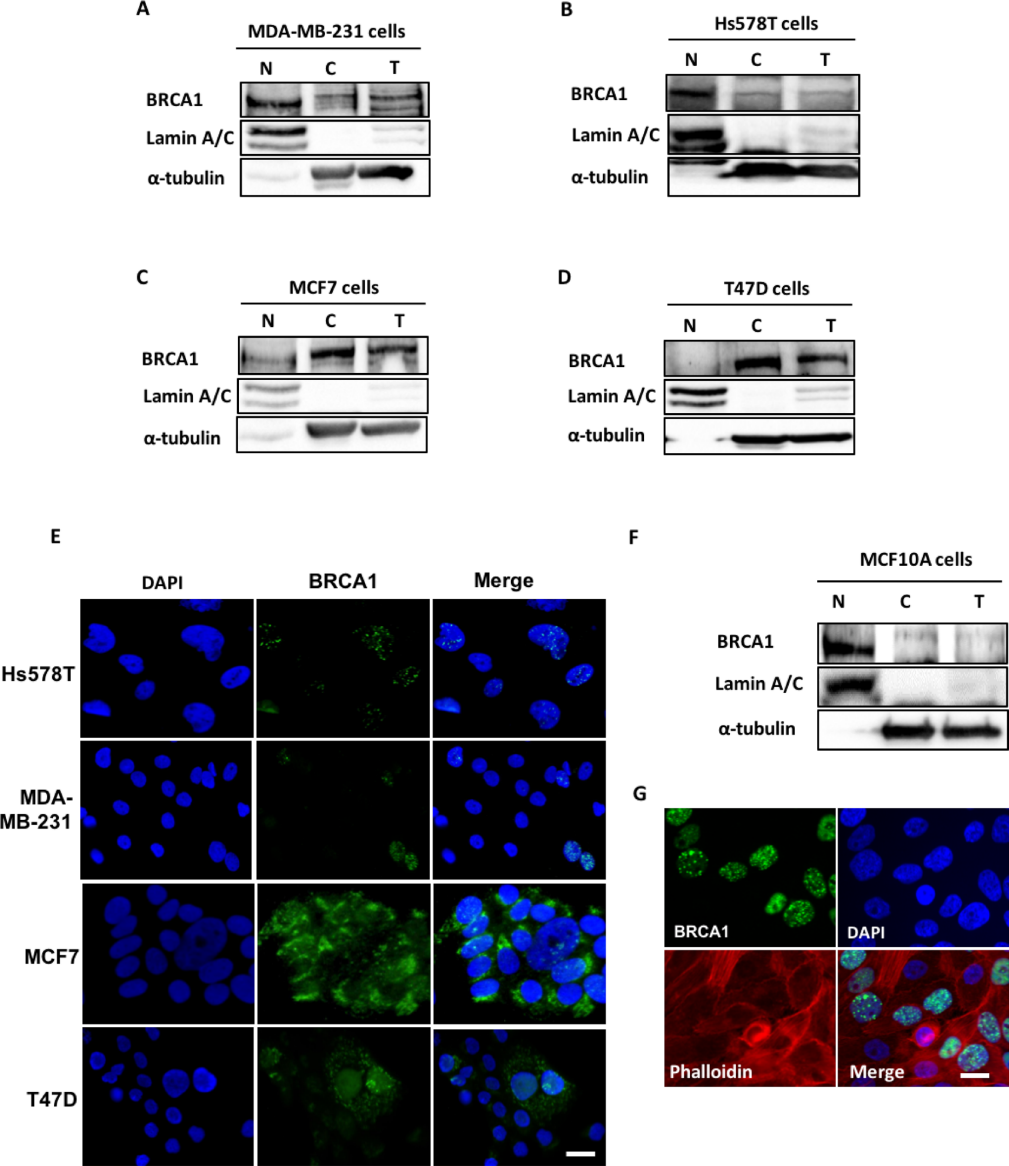
BRCA1 is predominantly cytoplasmic in ER-positive breast cancer cells (**A**–**D**) Nuclear (N), cytoplasmic (C) and whole cell extracts (T) were prepared from ER-negative breast cancer cells **(**A) MDA MB-231 and (B) Hs578T, the ER-positive (C) MCF7 and (D) T47D breast cancer cells, as well as a (**F**) normal mammary epithelial cell line MCF10A. Protein extracts were resolved by SDS-PAGE gel and immunoblotted with antibodies against BRCA1, Lamin A/C and α-tubulin. Lamin A/C and α-tubulin were used to loading controls for the nuclear and cytoplasmic fractions respectively. Representative images of immunofluorescence staining for BRCA1 localisation in (**E**) Hs578T, MDA-MB-231, MCF7 and T47D breast cancer cells and (**G**) normal mammary epithelial cells MCF10A are shown. The cells were labelled with anti-BRCA1 (Ab-1) antibody and the signal was detected with green fluorescent Alexa Fluor^®^ 488 anti–Mouse IgG. The nuclei were counterstained with blue fluorescent 4′,6-diamidino-2-phenylindole (DAPI). Cells were visualised under Zeiss Axio Vert A1 inverted microscope (Zeiss) and the images were captured with a QIClick CCD Camera (Q Imaging) and processed with Q-capture pro software. Scale bar represents 50 µm.

The data showing that BRCA1 physically binds ACCA, which is a cytoplasmic protein [10–12], suggest that BRCA1 has metabolic roles that requires it to be localised to the cytoplasm. However, there has been conflicting reports regarding the subcellular localisation of the BRCA1 protein in breast cancer cells [4, 26]. To study the sub-cellular localisation of BRCA1, we selected two ER-positive breast cancer cell line models (MCF7 and T47D), two ER-negative breast cancer cell lines (MDA-MB-231 and Hs578T) and a normal breast cell line (MCF10A) for analysis. We found that BRCA1 was predominantly localised in the nuclei of the ER-negative MDA MB 231 and Hs578T cells using subcellular fractionation method (Figure 3A and 3B). In contrast, BRCA1 was predominantly localised in the cytoplasm of MCF7 and T47D breast cancer cells which are ER-positive (Figure 3C and 3D). This localisation pattern was confirmed using immunofluorescence (Figure 3E). In a normal mammary epithelial cell line MCF10A however, BRCA1 was predominantly nuclear (Figure 3F and 3G). To demonstrate the relevance of cytoplasmic BRCA1 in cell metabolism, we confirmed the association between endogenous BRCA1 and phosphorylated ACCA in MCF7 and T47D breast cancer cells using co-immunoprecipitation. As expected, p-ACCA (S^79^) co-precipitated with BRCA1 and reciprocally, BRCA1 co-precipitated with p-ACCA (S^79^) in both MCF7 and T47D breast cancer cells (Supplementary Figure 2A and 2B). Control IgG did not yield any interactions.

### IGF-I induces dephosphorylation of ACCA and increases FASN abundance in ER-positive breast cancer cell lines

**Figure 4:**
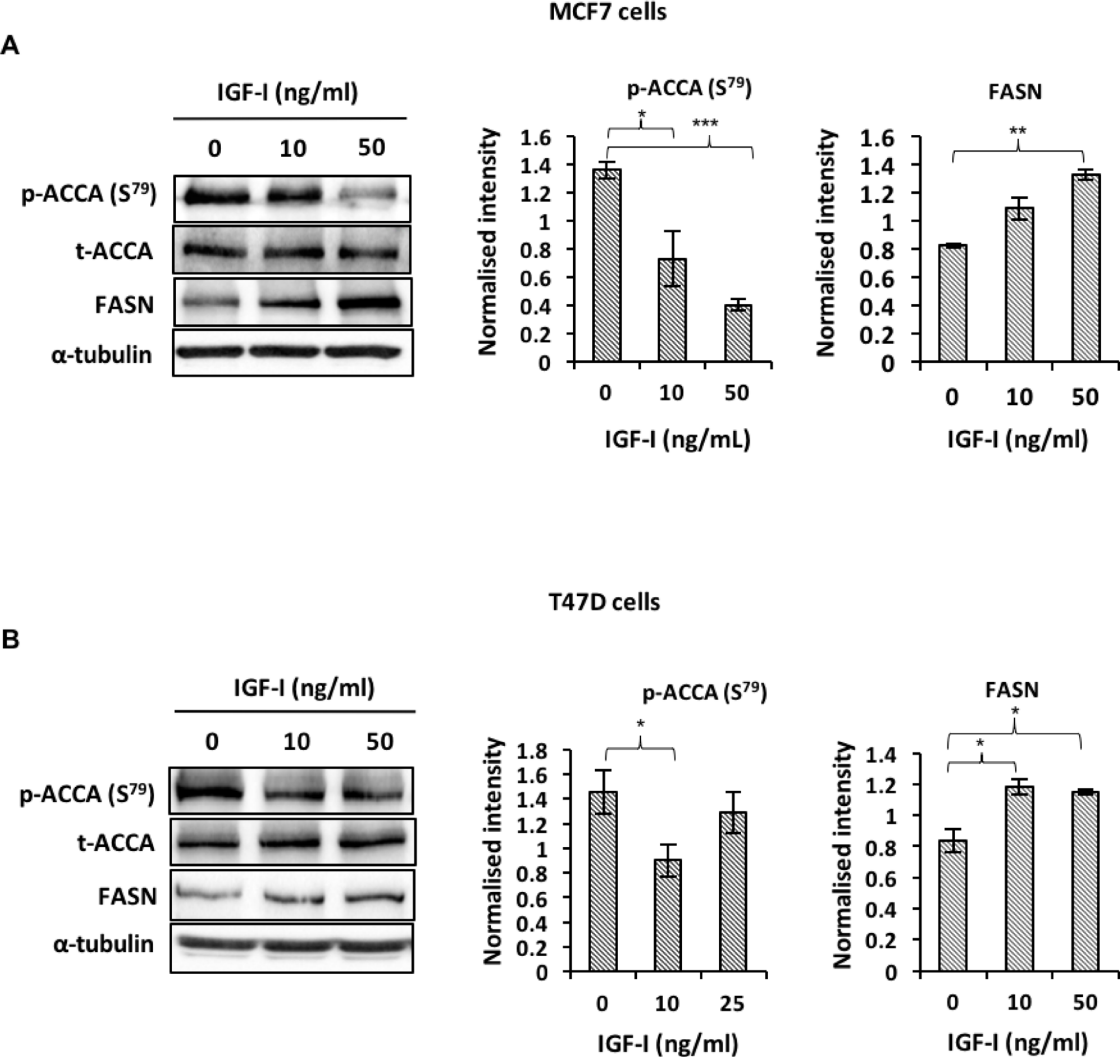
IGF-I affects fatty acid synthesis by inducing de-phosphorylation of ACCA and increasing FASN abundance in ER-positive breast cancer cell lines Western blot analysis of (**A**) MCF 7 and (**B**) T47D cells treated with 0, 10 and 50 ng/ml IGF-I for 48 hours. Representative blots of each protein from at least three experiments are shown. The graphs show densitometry analyses of p-ACCA (S^79^) normalized to total ACCA and FASN normalized to α-tubulin. Each bar represents mean ± S.E.M. of three independent experiments. ^*^P < 0.05, ^**^P < 0.01 and ^***^p < 0.001, one-way ANOVA.

Next, we investigated how IGF-I may regulate key enzymes of the fatty acid synthesis pathway. ACCA, which catalyses a rate-limiting step of the fatty acid synthesis pathway, is activated by dephosphorylation on serine-79 residues and produces substrates for FASN [15, 16, 27]. In both MCF7 and T47D cells, our data show that IGF-I induces dephosphorylation of ACCA and upregulates FASN downstream of ACCA (Figure 4A and 4B). These data suggest that IGF-I favours fatty acid synthesis by promoting the active state of ACCA and increasing FASN abundance.

### IGF-I induces dephosphorylation of ACCA by reducing the interaction between BRCA1 and p-ACCA (S^79^)

**Figure 5:**
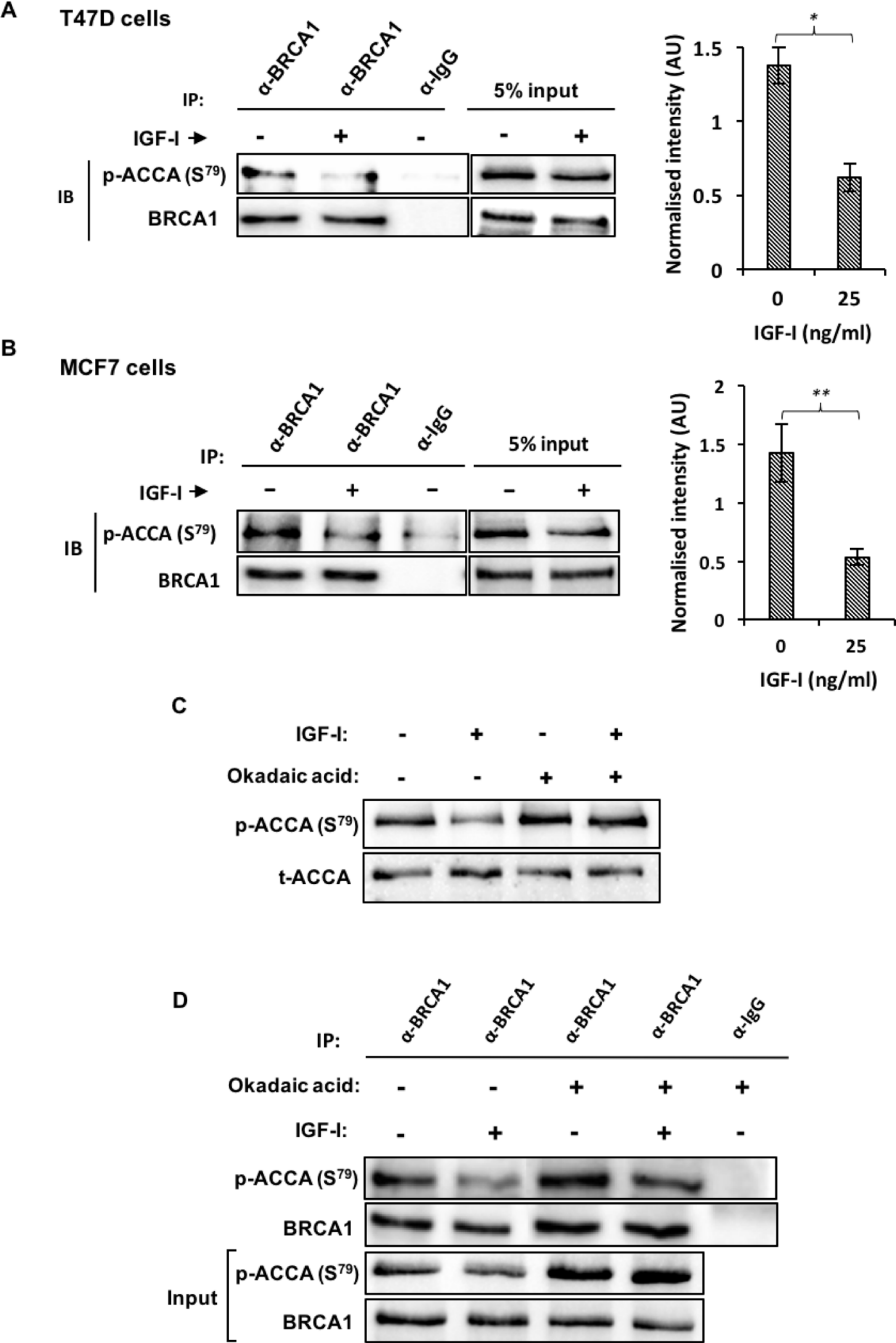
IGF-I reduces the interaction between BRCA1 and p-ACCA (S^79^) in MCF7 and T47D ER-breast cancer cells (**A**) T47D and (**B**) MCF7 cells were stimulated with 25 ng/l of IGF-I for 30 minutes. The lysates were immunoprecipitated with 2 μg anti-BRCA1 antibody or negative control IgG, followed by western blotting using BRCA1 and p-ACCA (S^79^) antibodies. Inputs represent lysates not used for immunoprecipitation. P-ACCA (S^79^) signal intensity was normalized to BRCA1 and presented graphically besides the representative blots. Three independent experiments were conducted and the data is presented as mean ± SE. ^*^p < 0.05, ^**^p < 0.01 and ^***^p < < 0.001, Student’s *t*-test (IGF-I versus control). (**C**, **D**) MCF7 cells were pre-incubated with (+) or without (−) okadaic acid (1 μM) for 30 minutes and spiked with either serum-free media (−) or 25 ng/ml IGF-I (+) for an additional 30 minutes. (C) Representative blots of p-ACCA (S^79^) and total ACCA are shown. (D) Lysates were subjected to immunoprecipitation with either anti-BRCA1 antibody or isotype-matched negative control IgG and immunoblotted with BRCA1 and p-ACCA (S^79^) antibodies. Input represent lysates not used for immunoprecipitation. Representative blots from three independent experiments are shown.

Our aforementioned data show that IGF-I dephosphorylates ACCA in luminal breast cancer cell lines which are known to express full-length, wildtype BRCA1 [28, 29]. Since BRCA1 regulates fatty acid synthesis by preventing the dephosphorylation of ACCA [10], these data then suggest that IGF-I somehow circumvents the protective role of BRCA1. We therefore hypothesised that IGF-I induces dephosphorylation of ACCA by reducing the interaction between BRCA1 and p-ACCA (S^79^). To study the impact of IGF-I on the association between BRCA1 and p-ACCA (S^79^), lysates from MCF7 and T47D cells treated with IGF-I were subjected to immunoprecipitation using a BRCA1 antibody and analysed using western blotting. Compared with control, IGF-I reduced the amount of p-ACCA (S^79^) co-precipitating with BRCA1 in both MCF7 and T47D breast cancer cell lines (Figure 5A and 5B). The interaction between BRCA1 and p-ACCA (S^79^) has previously been shown to be dependent on the level of ACCA phosphorylation [10]. Since we have also shown that IGF-I reduces ACCA phosphorylation, a caveat was whether the observed changes were secondary to ACCA dephosphorylation. To rule out this possibility, we used okadaic acid, a protein phosphatase inhibitor to inhibit dephosphorylation of ACCA by IGF-I. As expected, the IGF-I-induced dephosphorylation of ACCA was effectively inhibited in the presence of okadaic acid (Figure 5C). This inhibition ensured that the level of ACCA phosphorylation was similar between cells treated with IGF-I and the control. Using this approach, our immunoprecipitation data showed that IGF-I reduced the association between BRCA1 and p-ACCA (S^79^) even when ACCA dephosphorylation was inhibited (Figure 5D). This suggests that the reduction in the association between BRCA1 and p-ACCA (S^79^) is independent of the abundance of phosphorylated ACCA, ruling out the possibility that the reduction in the association is secondary to ACCA dephosphorylation.

### BRCA1 silencing enhances the effects of IGF-I in MCF7 breast cancer cells

**Figure 6:**
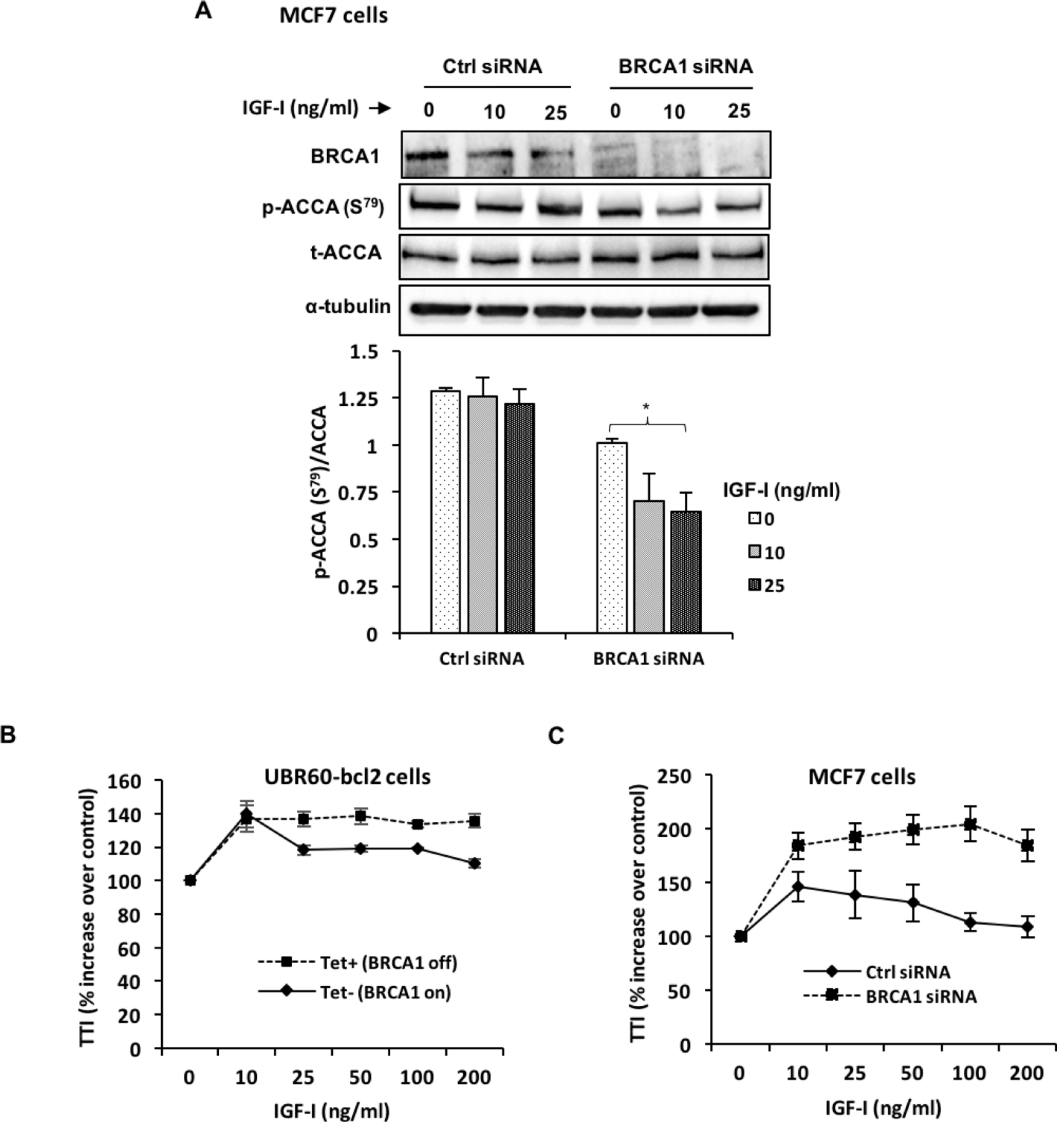
BRCA1 silencing enhances IGF-I signaling and effects on ACCA (**A**, **B**) MCF7 breast cancer cells were transfected with BRCA1 siRNA or control siRNA for 48 hours before stimulation with 25 ng/ml IGF-I for 30 minutes. (A) Representative blots of western blotting analysis of p-ACCA (S^79^) and total ACCA are shown. The graph shows densitometry analyses of p-ACCA (S^79^) normalized to total ACCA. Each bar represents mean ± S.E.M. of three independent experiments. ^*^p < 0.05, ^**^p < 0.01 and ^***^p < 0.001, one-way ANOVA. (B) UBR60-bcl2 cells with or without 1 mg/ml tetracycline and (**C**) MCF7 cells transfected with control siRNA or BRCA1 siRNA were treated with increasing doses (0–200 ng/ml) of IGF-I for 24 hours. The cells were pulsed with [3H]-labelled thymidine (1 μCi) 4 hours before the end of the experiment. The radioactive label was precipitated with trichloracetic acid, solubilised with sodium hydroxide and the signal was detected using liquid scintillation counter. The experiments were repeated 3 times and results are expressed as percentage increase for treatment over control. The graphs represent mean ± S.E.M. of three independent experiments.

Several studies have shown that IGF-I and the IGF-IR are upregulated in the absence of BRCA1 in different *in vitro* and *in vivo* models [30–32]. We sought to investigate whether BRCA1 loss might also enhance the effects of IGF-I in our model. Surprisingly, silencing BRCA1 only modestly enhanced the phosphorylation of the IGF-IR by IGF-I (Supplementary Figure 3A). Interestingly, we also observed a moderate reduction in the total levels of the IGF-IR in response to IGF-I when BRCA1 was silenced, which was not observed under non-silencing conditions. Downstream of the IGF-IR however, the activation of AKT phosphorylation by IGF-I was higher when BRCA1 was silenced compared to control siRNA (Supplementary Figure 3B). Next, we asked whether BRCA1 silencing could also enhance ACCA dephosphorylation induced by IGF-I. For this, we silenced BRCA1 in the MCF7 cell line and treated these cells with IGF-I for 30 minutes. In cells transfected with control siRNA, IGF-I moderately induced de-phosphorylation of ACCA, but this effect was significantly enhanced in the cells transfected with BRCA1 siRNA (Figure 6A).

We also further investigated the role of BRCA1 in regulating the proliferative response of cells to IGF-I. In the UBR60-bcl2 cells with tetracycline-regulated BRCA1 expression [25], *BRCA1* expression was repressed by tetracycline (Tet+, BRCA1 off) and induced by tetracycline withdrawal (Tet-, BRCA1 on). The cells were then stimulated with increasing doses of IGF-I for 24 hours. IGF-I induced cell proliferation under both conditions, however, the cells with repressed BRCA1 displayed an increased and sustained response to IGF-I, especially at higher doses when compared with cells overexpressing BRCA1 (Figure 6B). Next, we sought to replicate these observations in a breast cancer cell line. For this, MCF7 cells transfected with BRCA1 siRNA and control siRNA were incubated with increasing doses of IGF-I for 24 hours. Similar to UBR60-bcl2 cell line, MCF7 cells with siRNA-mediated BRCA1 repression displayed an enhanced response to IGF-I which was sustained at all doses of IGF-I in contrast to control siRNA (Figure 6C).

## DISCUSSION

Until recently, all the known cellular functions of BRCA1 were linked to the genome [6–8]. In the background of higher disease penetrance among BRCA1/2 mutation carriers in recent cohorts compared to older cohorts with the same genetic predisposition, lifestyle factors have been associated with a significant impact on disease onset [2, 33]. These observations appear to link BRCA1 with roles beyond the genome such as in the regulation of metabolic status.

Here we report that BRCA1 plays a key role in lipogenesis by regulating ACCA phosphorylation and FASN abundance downstream of ACCA. An increase in ACCA phosphorylation following BRCA1 induction in UBR60-bc2 cells is consistent with the data showing that BRCA1 binds the phosphorylated and inactive form of ACCA [11, 12], protecting it from dephosphoryation. The association between BRCA1 and phosphorylated ACCA restrains endogenous fatty acids synthesis [10] and this effect is further reflected by a reduction in FASN abundance downstream of ACCA in our UBR60 cell model. Similarly, siRNA-mediated reduction of BRCA1 in breast cancer cells resulted in a decrease in ACCA phosphorylation and an increase in FASN abundance. Since BRCA1 acts as a brake on lipogenesis by binding phosphorylated ACCA, the loss of BRCA1 is consistent with releasing this brake and allowing synthesis of long chain saturated fatty acids. A similar effect has been demonstrated in human adipose tissue. In pre-adipocytes, increased BRCA1 expression correlated with an increase in ACCA phosphorylation and FASN downregulation to inhibit fatty acid synthesis [34]. In contrast however, *BRCA1* expression was reduced during adipogenesis, resulting in decreased ACCA phosphorylation and an increase in FASN to allow lipogenesis [34]. Cancer cells typically exhibit lipogenic phenotype characterised by upregulation of lipogenic enzymes and increased endogenous synthesis of fatty acids [35]. Our data showing that BRCA1 regulates ACCA phosphorylation and FASN abundance suggests that the impairment of this function by germline mutations or by epigenetic events may favour carcinogenesis. It has been shown that decreased BRCA1 in skeletal muscle resulted in increased storage of intracellular lipid and reduced insulin signalling [36]. In addition, an imaging spectroscopy study examining metabolite levels in breast tissue of women with BRCA mutations but without evidence of cancer, identified changes in lipid levels [37]. These studies indicate a normal role for BRCA1 in regulating lipid metabolism in tissues and specifically in the breast. In light of our data, we believe it would be interesting in future work to assess additional metabolic changes, such as altered metabolic products as a consequence of BRCA1 dysfunction.

Shortly after BRCA1 was cloned in 1994, early research into the *BRCA1* gene product suggested it was an exclusively nuclear protein with little or no known cytoplasmic functions [38]. At the time, the maintenance of genome integrity was the most described and widely accepted role of BRCA1, therefore the cytoplasmic localisation of BRCA1 reported in several studies [39, 40] was incompatible with this function. The recently discovered functions of BRCA1, such as in fatty acid synthesis, clearly dictate that BRCA1 should be localised in the cytoplasm to carry out these functions. The association between BRCA1 and phosphorylated ACCA reported by others [10, 11] and confirmed in our study, also suggests that BRCA1 would have to be cytoplasmic for this interaction to occur. The cytoplasmic localization of BRCA1 was also confirmed in clinical specimens in a study of 103 women with breast cancer, where cytoplasmic localisation of BRCA1 was found in 51.4% of tumours and exclusive nuclear localisation was only found in a minority of cases [41].

Our study is the first to demonstrate a clearly defined localisation pattern between cell line models of ER-positive and ER-negative breast cancer subtypes. Although the reason for this novel distribution pattern is currently unclear, it is possible that it reflects the intrinsic molecular subtypes of breast tumours. More importantly, this localisation pattern suggests that besides technical differences, biological heterogeneity may also underlie the discrepant data on BRCA1 localisation in unselected samples in different studies [3, 4, 39, 42].

Clinically, our data is consistent with the findings that cytoplasmic BRCA1 is a feature of less aggressive ER-positive breast cancers with good prognosis [39, 40]. Interestingly, the ER-positive breast cancer cell lines used in this study typically display less aggressive phenotypes when compared to ER-negative breast cancer cells [43, 44]. However, a more recent study reported no prognostic value of BRCA1 localisation, but restriction of the analysis to patients aged >40 years revealed an association between cytosolic BRCA1 and metastasis [45]. In the Santivasi *et al.* study however, unlike in the other studies, cytoplasmic BRCA1 was reported to be a marker of poor prognosis. The reasons for this conflicting prognostic value of BRCA1 localisation are unknown and warrant further investigation.

ACCA catalyses the rate limiting step of the fatty acid synthesis pathway and has been shown to be important to the survival of cancer cells [24, 46, 47]. Phosphorylation reduces ACCA activity by reducing its V_*max*_ and sensitivity to allosteric activation by citrate [47], therefore our data showing that IGF-I induces ACCA dephosphorylation suggests that IGF-I sensitises ACCA to activation. Additionally, Ser-79 is a critical phosphorylation site for ACCA deactivation, therefore the effects of IGF-I on ACCA reported here are consistent with ACCA activation. Early studies have shown that insulin also activates ACCA by reducing its phosphorylation [16, 48]. Despite belonging to the insulin family, very few studies have demonstrated the effects of IGF-I on ACCA phosphorylation. Recently, IGF-I has been shown to reduce ACCA phosphorylation in HCT-8 colorectal cancer cells, however, IGF-I also reduced the abundance of total ACCA, resulting in no net change in ACCA activity or lipid synthesis [49].

Our group has previously published that downstream of ACCA, IGF-I increases FASN abundance in cells expressing full-length, wildtype BRCA1 and that the cells are dependent on FASN for their proliferative response [23]. The current report presents a novel mechanism by which IGF-I induces ACCA dephosphorylation. The disruption of the physical association between BRCA1 and phosphorylated ACCA has been suggested to sensitise ACCA to dephosphorylation [10]. Our co-immunoprecipitation studies show that IGF-I disrupts this interaction, rendering ACCA susceptible to dephosphorylation. While we demonstrate that IGF-I reduces the interaction between BRCA1 and p-ACCA (S^79^), we did not investigate the precise mechanism in this study. However, available evidence show that BRCA1 can also be phosphorylated at multiple sites and this phosphorylation is important for its function [50], including its interaction with other proteins. Martin and Ouchi, 2005 have shown that BRCA1 interacts with X-linked inhibitor of apoptosis protein (XIAP) in an ovarian carcinoma cell line SNU-251 and that the BRCA1-XIAP complex is disrupted by UV-induced phosphorylation of BRCA1 [51]. In another study, IGF-I induced BRCA1 phosphorylation in an AKT-dependent manner in breast and ovarian cancer cell lines [52]. We therefore hypothesize that IGF-I may be inducing BRCA1 phosphorylation in our model, resulting in the dissociation of the BRCA1-p-ACCA complex. Our future studies will further elucidate this mechanism.

High circulating levels of IGF-I increase the risk of breast cancer in women from families with *BRCA 1/2* mutations [53] and BRCA1 deficiency has been linked to an increase in IGF-I in both *in vitro* and *in vivo* models [22, 30]. In addition to these observational studies, investigations of genetic determinants of breast cancer risk in BRCA1 mutation carriers have identified associations with genetic variants in the IGF-IR [54] and IGF-II [55], indicating that the IGF-pathway is causally related to breast cancer development in women with BRCA1 mutations. In this study, we further studied the impact of BRCA1 deficiency on the action of excess IGF-I. Our data also show that in addition to dissociation of the BRCA1-p-ACCA (S^79^) complexes, siRNA-mediated loss of BRCA1 also renders ACCA susceptible to dephosphorylation. This is demonstrated by the enhanced ACCA dephosphorylation by IGF-I when BRCA1 was silenced. All the observed alterations in lipogenic enzymes are consistent with increased *de novo* fatty acid synthesis, which is supportive to cell growth. Proliferating cells are dependent on fatty acids for energy and synthesis of biological membranes [56, 57]. Consistent with this notion, breast cancer cells have been shown to synthesise up to 95% of their fatty acids regardless of exogenous supply and this is facilitated by upregulation of activity and abundance of fatty acid enzymes including ACCA and FASN [58].

BRCA1 knockdown also enhanced the effects of exogenous IGF-I on AKT as expected, but to our surprise, the phosphorylation of the IGF-IRß upstream of AKT was slightly reduced. The observed reduction in IGF-IRß phosphorylation in response to IGF-I may be due to ligand-induced receptor internalisation which has been shown to sustain AKT phosphorylation [59]. The internalised IGF-I receptors can be recycled to the membrane and this process is more energy-efficient than *de novo* receptor synthesis [59]. However, BRCA1 deficiency may impair receptor recycling in our model, which may then be over-compensated for by synthesis of new receptor molecules. In spite of this, a longer time-point may be necessary to observe a high steady-state receptor turnover in response to IGF-I. Further studies are required to understand the effects of BRCA1 deficiency on IGF-I receptor recycling.

Our group has previously shown that the induction of a negative regulator of the IGF-IR/AKT pathway: PTEN, desensitises cells to high doses of IGF-II, but IGFBP-2, when independent from IGF-II can restore the response to high doses by suppressing PTEN [60]. In this study, our data show that BRCA1 deficiency increases the cellular proliferative response to IGF-I and that cells remain sensitised to IGF-I at high doses. These observed effects may be mediated through the mechanism demonstrated for IGF-II in the Perks *et al.* study. Additionally, we have previously shown that loss of PTEN is associated with IGFBP-2 expression [61]. Remarkably, we also observed that BRCA1 deficiency in this study upregulated IGFBP-2 and PTEN was supressed (data not shown). Taken together, these data suggest that BRCA1 restrains IGF-I-induced cell proliferation but when this restraint is lost due to BRCA1 deficiency, cells proliferate unrestrained partly due to the loss of PTEN and upregulated *de novo* fatty acid synthesis.

## CONCLUSIONS

We report that BRCA1 restrains IGF-I induced cell proliferation and that this function is dependent on the physical association between BRCA1 and phosphorylated ACCA in the cytoplasm of ER-positive breast cancer cells. Any cellular event that reduces or abolishes this interaction such as IGF-I action or BRCA1 deficiency, upregulates fatty acid synthesis and supports cell growth. The model presented here suggests an opportunity to target the lipogenic pathway in BRCA1-related breast cancers.

## MATERIALS AND METHODS

### Reagents

Recombinant, human IGF-I peptide was purchased from Gropep (Adelaide, South Australia, Australia). Okadaic acid was purchased from Calbiochem (San Diego, CA, USA).

### Cell culture

Human breast cancer cells, MCF-7, T47D, MDA-MB 231 and Hs578T were purchased from the European Collection of Authenticated Cell Cultures (ECACC) and maintained as previously published [23]. UBR60-bcl2 cell line was a generous gift from Professor Paul Harkin (Queen’s University of Belfast, Ireland). The UBR60-bcl2 cell line expresses BRCA1 under the control of tetracycline-regulated promoter and has previously been described elsewhere [25].

### siRNA and transfections

Small interfering RNA (siRNA) targeting BRCA1 were acquired from Qiagen (Manchester, UK) and the sequences are as follows; siRNA1 (Hs_siRNA_9): 5′AACCTATCGGAAGAAGGCAAG-3′ and siRNA2 (Hs-siRNA_14): 5′-CAGGAAATGGCTGAACTAGAA-3′. All stars negative control siRNA (Qiagen, Manchester, UK) with no target in the human genome was used as control. Transient transfection of siRNA was performed using Saint-Red Transfection Reagent (Synvolux Therapeutics, Groningen, Netherlands) according to the manufacturer’s instructions. Western blotting was used to validate knockdown efficiency.

### Western blotting

Cells were either lysed directly on culture plates and scraped or trypsinised first before lysis using cell lysis buffer described elsewhere [62]. Equal amount of proteins from lysates were separated on sodium dodecyl sulfate-polyacrylamide gel electrophoresis (SDS–PAGE) and transfered onto supported nitrocellulose membranes (BioRad, Hertfordshire, UK). Non-specific binding were eliminated by incubating membranes with 1% w/v milk in tris- buffered saline Tween-20 (TBST) overnight. Membranes were then probed with the following antibodies according to the manufacturer’s recommended dilutions; ACCA (Cell Signaling, Danvers, MA, USA), p-ACCA (S^79^) (Cell Signaling, Danvers, MA, USA), FASN (BD Biosciences, Franklin Lakes, NJ, USA), IGF-IR (Cell Signaling, MA, USA), p-IGF-IR (Y^1135/1136^) (Cell Signaling, Danvers, MA, USA), AKT (Cell Signaling, Danvers, MA, USA) and p-AKT (S^473^) (Cell Signaling, Danvers, MA, USA). Membranes were washed and incubated with anti-mouse IgG HRP or anti-rabbit IgG HRP secondary antibodies. Membranes were washed and the signals were detected using SuperSignal West Dura and Femto Chemiluminescent Substrates (Thermo Scientific, Rockford, IL, USA). The bands were analysed using Image J (National Institutes of Health, Bethesda, Maryland, USA).

### Tritiated thymidine incorporation assay

Cells were seeded in 24-well plates at 0.025 × 10^6^ cells per well in growth media and maintained for 24 hrs in 5% CO_2_ at 37° C. Cells were then serum-starved for 24 hours before dosing with 0, 10, 25, 50, 100 and 200 ng/ml IGF-I in serum-free medium for 24 hours. Each IGF-I dose was performed in triplicate. In the last 4 hours of the experiment, cells were incubated with 0.1 µCi [^3^H]-Thymidine (Perkin Elmer, Boston, MA) per well. After 4 hours, cells were washed with 5% w/v Trichoroacetic acid (TCA) (Sigma-Aldrich, St Louis, MO, USA) solution for 10 minutes at 4° C. TCA was then removed and cells were treated with 1M NaOH (Fisher Scientific Ltd, Leicestershire, UK) for 1 hour at room temperature. Scintillation fluid (Perkin Elmer, Boston, MA) was added and radioactivity was measured in a Liquid Scintillation Analyzer (Perkin Elmer, Boston, MA). Data was recorded as disintegration per minute (DPM).

### Co-immunoprecipitation

Cells were lysed in cell lysis buffer supplemented with protease inhibitors and phosphatase inhibitor cocktail (Sigma-Aldrich, St Louis, MO, USA). Equal amounts of protein (1 mg) from the resulting lysates were incubated with 2 µg of anti-BRCA1 (Merck Millipore, Billerica, MA, USA), anti-p-ACCA (S^79^) (Cell Signaling, Danvers, MA, USA) and negative control IgG (Dako, Glostrup, Denmark) antibodies overnight at 4° C. Lysates were then incubated with 10 ug affinity purified secondary antibody (Merk-Millipore, Billerica, MA, USA) for 1 hour, followed by incubation with Protein A/G PLUS immunoprecipitation reagent (Santa Cruz, Dallas, Texas, USA) for 1 hour at 4° C. After centrifugation and washing with lysis buffer, immune complexes were eluted by heating with sample loading buffer (Sigma-Aldrich, St Louis, MO, USA) for 5 minutes at 95° C and analysed by western blotting.

### Subcellular fractionation

Extraction of cytoplasmic and nuclear protein fractions was achieved using a NE-PER Nuclear and Cytoplasmic Extraction Kit (Thermo Scientific, Rockford, IL USA). Whole cell fractions were obtained by routine whole cell lysis using cell lysis buffer. Protein concentrations were determined using Pierce BCA Protein Assay (Thermo Scientific, Rockford, IL USA) and equal amounts of cytosolic, nuclear and whole cell extracts were analysed by western blotting.

### Immunofluorescence

Cells cultured on 22 × 22 mm coverslips were fixed with 4% paraformaldehyde for 20 minutes. Fixed cells were permeabilized using 0.5% Triton-X for 15 minutes and non-specific binding was blocked with 3% BSA in PBS for 1 hour. For immunostaining, cells were incubated with BRCA1 antibody (1:500) for 1 hour at room temperature. After washing three times with PBS, cells were incubated with secondary antibody for 1 hour at room temperature. After washing three times with PBS, slides were mounted and counterstained using Vectashield^®^ Mounting Medium with DAPI (Vecta laboratories, H-1200). Slides were then visualised on a Zeiss Axio Vert A1 inverted microscope and images were captured with a QIClick CCD Camera (Q Imaging) interfaced with Q-capture Pro software.

### Statistical analyses

Data were analysed using IBM SPSS statistics 23 (IBM Corporation, version 23.0.0.2) and presented as mean ± S.E.M of a minimum of three independent experiments. Independent samples *t*-test was used to compare means of two groups and for the means of more than two groups, one-way analysis of variance (ANOVA) was used followed by a Bonferroni Multiple Comparison Test. For both tests, a *p*-value of equal or less than 0.05 was considered statistically significant.

## SUPPLEMENTARY MATERIALS FIGURES



## Abbreviations

ACCA: Acetyl CoA carboxylase a
BRCA: Breast cancer 1, early onset
ECACC: European collection of authenticated cell cultures
ER: Estrogen receptor
FASN: Fatty acid synthase
IGF-I: Insulin-like growth factor 1
IGF-IR: Insulin-like growth factor 1 receptor
SDS-PAGE: Sodium dodecyl sulfate-polyacrylamide gel electrophoresis
SiRNA: Short interfering RNA
TBST: Tris-buffered saline Tween-20
TCA: Trichoroacetic acid

## References

[1] Miki Y, Swensen J, Shattuck-Eidens D, Futreal PA, Harshman K, Tavtigian S, Liu Q, Cochran C, Bennett LM, Ding W, Bell R, Rosenthal J, Hussey C (1994). A strong Candidate for the Breast and Ovarian Cancer. Science.

[2] King MC, Marks JH, Mandell JB (2003). Breast and ovarian cancer risks due to inherited mutations in BRCA1 and BRCA2. Science.

[3] Yoshikawa K, Honda K, Inamoto T, Shinohara H, Yamauchi A, Suga K, Okuyama T, Shimada T, Kodama H, Noguchi S, Gazdar AF, Yamaoka Y, Takahashi R (1999). Reduction of BRCA1 Protein Expression in Japanese Sporadic Breast Carcinomas and Its Frequent Loss in BRCA1- associated Cases. Clin Cancer Res.

[4] Wilson CA, Ramos L, Villaseñor MR, Anders KH, Press MF, Clarke K, Karlan B, Chen JJ, Scully R, Livingston D, Zuch RH, Kanter MH, Cohen S (1999). Localization of human BRCA1 and its loss in high-grade, non-inherited breast carcinomas. Nat Genet.

[5] Lambie H, Miremadi A, Pinder SE, Bell JA, Wencyk P, Paish EC, Macmillan RD, Ellis IO (2003). Prognostic significance of BRCA1 expression in sporadic breast carcinomas. J Pathol.

[6] Bae I, Fan S, Meng Q, Rih JK, Kim HJ, Kang HJ, Xu J, Goldberg ID, Jaiswal AK, Rosen EM (2004). BRCA1 Induces Antioxidant Gene Expression and Resistance to Oxidative Stress. Cancer Res.

[7] Moynahan ME, Chiu JW, Koller BH, Jasin M (1999). Brca1 Controls Homology-Directed DNA Repair. Mol Cell.

[8] Ruffner H, Verma IM (1997). BRCA1 is a cell cycle-regulated nuclear phosphoprotein. Proc Natl Acad Sci USA.

[9] Nkondjock A, Ghadirian P (2007). Diet quality and BRCA-associated breast cancer risk. Breast Cancer Res Treat.

[10] Moreau K, Dizin E, Ray H, Luquain C, Lefai E, Foufelle F, Billaud M, Lenoir GM, Venezia ND (2006). BRCA1 affects lipid synthesis through its interaction with acetyl-CoA carboxylase. J Biol Chem.

[11] Magnard C, Bachelier R, Vincent A, Jaquinod M, Kieffer S, Lenoir GM, Venezia ND (2002). BRCA1 interacts with acetyl-CoA carboxylase through its tandem of BRCT domains. Oncogene.

[12] Sheng Y, Tong L (2008). Structural evidence for direct interactions between the BRCT domains of human BRCA1 and a phospho-peptide from human ACC1. Biochemistry.

[13] Berod L, Friedrich C, Nandan A, Freitag J, Hagemann S, Harmrolfs K, Sandouk A, Hesse C, Castro CN, Bähre H, Tschirner SK, Gorinski N, Gohmert M (2014). De novo fatty acid synthesis controls the fate between regulatory T and T helper 17 cells. Nat Med.

[14] Widmer J, Fassihi KS, Schlichter SC, Wheeler KS, Crute BE, King N, Nutile-McMenemy N, Noll WW, Daniel S, Ha J, Kim KH, Witters LA (1996). Identification of a second human acetyl-CoA carboxylase gene. Biochem J.

[15] Ray H, Suau F, Vincent A, Dalla Venezia N (2009). Cell cycle regulation of the BRCA1/acetyl-CoA-carboxylase complex. Biochem Biophys Res Commun.

[16] Ha J, Daniel S, Broyles SS, Kim KH (1994). Critical phosphorylation sites for acetyl-CoA carboxylase activity. J Biol Chem.

[17] Fullerton MD, Galic S, Marcinko K, Sikkema S, Pulinilkunnil T, Chen ZP, O’Neill HM, Ford RJ, Palanivel R, O’Brien M, Hardie DG, Macaulay SL, Schertzer JD (2013). Single phosphorylation sites in Acc1 and Acc2 regulate lipid homeostasis and the insulin-sensitizing effects of metformin. Nat Med.

[18] Clark MA, Perks CM, Winters ZE, Holly JMP (2005). DNA damage uncouples the mitogenic response to IGF-I in MCF-7 malignant breast cancer cells by switching the roles of PI3 kinase and p21 WAF1/Cip1. Int J Cancer.

[19] Chen W, Wang S, Tian T, Bai J, Hu Z, Xu Y, Dong J, Chen F, Wang X, Shen H (2009). Phenotypes and genotypes of insulin-like growth factor 1, IGF-binding protein-3 and cancer risk: evidence from 96 studies. Eur J Hum Genet.

[20] Bhargava R, Beriwal S, McManus K, Dabbs DJ (2011). Insulin-like growth factor receptor-1 (IGF-1R) expression in normal breast, proliferative breast lesions, and breast carcinoma. Appl Immunohistochem Mol Morphol.

[21] Schayek H, Haugk K, Sun S, True LD, Plymate SR, Werner H (2009). Tumor suppressor BRCA1 is expressed in prostate cancer and controls insulin-like growth factor I receptor (IGF-IR) gene transcription in an androgen receptor-dependent manner. Clin Cancer Res.

[22] Kang HJ, Yi YW, Kim HJ, Hong YB, Seong YS, Bae I (2012). BRCA1 negatively regulates IGF-1 expression through an estrogen-responsive element-like site. Cell Death Dis.

[23] Zeng L, Biernacka KM, Holly JMP, Jarrett C, Morrison AA, Morgan A, Winters ZE, Foulstone EJ, Shield JP, Perks CM (2010). Hyperglycaemia confers resistance to chemotherapy on breast cancer cells : the role of fatty acid synthase. Endocr Relat Cancer.

[24] Chajès V, Cambot M, Moreau K, Lenoir GM, Joulin V (2006). Acetyl-CoA Carboxylase α Is Essential to Breast Cancer Cell Survival. Cancer Res.

[25] Harkin DP, Bean JM, Miklos D, Song Y, Truong VB, Englert C, Christians FC, Ellisen LW, Maheswaran S, Oliner JD, Haber DA (1999). Induction of GADD45 and JNK / SAPK-Dependent Apoptosis following Inducible Expression of BRCA1. Cell.

[26] Rodriguez JA, Schüchner S, Au WWY, Fabbro M, Henderson BR (2004). Nuclear-cytoplasmic shuttling of BARD1 contributes to its proapoptotic activity and is regulated by dimerization with BRCA1. Oncogene.

[27] Yoon S, Lee MY, Park SW, Moon JS, Koh YK, Ahn YH, Park BW, Kim KS (2007). Up-regulation of acetyl-CoA carboxylase alpha and fatty acid synthase by human epidermal growth factor receptor 2 at the translational level in breast cancer cells. J Biol Chem.

[28] Saal LH, Gruvberger-Saal SK, Persson C, Lövgren K, Jumppanen M, Staaf J, Jönsson G, Pires MM, Maurer M, Holm K, Koujak S, Subramaniyam S, Vallon-Christersson J (2008). Recurrent gross mutations of the PTEN tumor suppressor gene in breast cancers with deficient DSB repair. Nat Genet.

[29] Elstrodt F, Hollestelle A, Nagel JHA, Gorin M, Wasielewski M, Van Den Ouweland A, Merajver SD, Ethier SP, Schutte M (2006). BRCA1 mutation analysis of 41 human breast cancer cell lines reveals three new deleterious mutants. Cancer Res.

[30] Shukla V, Coumoul X, Cao L, Wang RH, Xiao C, Xu X, Andò S, Yakar S, Leroith D, Deng C (2006). Absence of the full-length breast cancer-associated gene-1 leads to increased expression of insulin-like growth factor signaling axis members. Cancer Res.

[31] Hudelist G, Wagner T, Rosner M, Fink-Retter A, Gschwantler-Kaulich D, Czerwenka K, Kroiss R, Tea M, Pischinger K, Köstler WJ, Attems J, Mueller R, Blaukopf C (2007). Intratumoral IGF-I protein expression is selectively upregulated in breast cancer patients with BRCA1/2 mutations. Endocr Relat Cancer.

[32] Liu B, Li D, Guan YF (2014). BRCA1 regulates insulin-like growth factor 1 receptor levels in ovarian cancer. Oncol Lett.

[33] Pijpe A, Manders P, Brohet RM, Collée JM, Verhoef S, Vasen HF, Hoogerbrugge N, van Asperen CJ, Dommering C, Ausems MG, Aalfs CM, Gomez-Garcia EB, Van’t Veer LJ, HEBON (2010). Physical activity and the risk of breast cancer in BRCA1/2 mutation carriers. Breast Cancer Res Treat.

[34] Ortega FJ, Moreno-Navarrete JM, Mayas D, García-Santos E, Gómez-Serrano M, Rodriguez-Hermosa JI, Ruiz B, Ricart W, Tinahones FJ, Frühbeck G, Peral B, Fernández-Real JM (2012). Breast cancer 1 (BrCa1) may be behind decreased lipogenesis in adipose tissue from obese subjects. PLoS One.

[35] Menendez JA, Lupu R (2007). Fatty acid synthase and the lipogenic phenotype in cancer pathogenesis. Nat Rev Cancer.

[36] Jackson KC, Gidlund EK, Norrbom J, Valencia AP, Thomson DM, Schuh RA, Neufer PD, Spangenburg EE (2014). BRCA1 is a novel regulator of metabolic function in skeletal muscle. J Lipid Res.

[37] Ramadan S, Arm J, Silcock J, Santamaria G, Buck J, Roy M, Leong KM, Lau P, Clark D, Malycha P, Mountford C (2015). Lipid and Metabolite Deregulation in the Breast Tissue of Women Carrying BRCA1 and BRCA2 Genetic Mutations. Radiology.

[38] Fabbro M, Rodriguez JA, Baer R, Henderson BR (2002). BARD1 induces BRCA1 intranuclear foci formation by increasing RING-dependent BRCA1 nuclear import and inhibiting BRCA1 nuclear export. J Biol Chem.

[39] Mylona E, Melissaris S, Nomikos A, Theohari I, Giannopoulou I, Tzelepis K, Nakopoulou L (2014). Effect of BRCA1 immunohistochemical localizations on prognosis of patients with sporadic breast carcinomas. Pathol Res Pract.

[40] Rakha EA, El-Sheikh SE, Kandil MA, El-Sayed ME, Green AR, Ellis IO (2008). Expression of BRCA1 protein in breast cancer and its prognostic significance. Hum Pathol.

[41] Wiener D, Gajardo-Meneses P, Ortega-Hernández V, Herrera-Cares C, Díaz S, Fernández W, Cornejo V, Gamboa J, Tapia T, Alvarez C, Carvallo P (2015). BRCA1 and BARD1 colocalize mainly in the cytoplasm of breast cancer tumors, and their isoforms show differential expression. Breast Cancer Res Treat.

[42] Chen Y, Chen CF, Riley DJ, Allred DC, Chen PL, Von Hoff D, Osborne CK, Lee WH (1995). Aberrant subcellular localization of BRCA1 in breast cancer. Science.

[43] Kao J, Salari K, Bocanegra M, Choi YL, Girard L, Gandhi J, Kwei KA, Hernandez-Boussard T, Wang P, Gazdar AF, Minna JD, Pollack JR (2009). Molecular profiling of breast cancer cell lines defines relevant tumor models and provides a resource for cancer gene discovery. PLoS One.

[44] Subik K, Lee JF, Baxter L, Strzepek T, Costello D, Crowley P, Xing L, Hung MC, Bongfiglio T, Hicks DG, Tang P (2010). The Expression Patterns of ER, PR, HER2, CK5 / 6, EGFR, Ki-67 and AR by Immunohistochemical Analysis in Breast Cancer Cell Lines. Breast Cancer (Auckl).

[45] Santivasi WL, Wang H, Wang T, Yang Q, Mo X, Brogi E, Haffty BG, Chakravarthy AB, Xia F (2015). Association between cytosolic expression of BRCA1 and metastatic risk in breast cancer. Br J Cancer.

[46] Brusselmans K, De Schrijver E, Verhoeven G, Swinnen JV (2005). RNA interference-mediated silencing of the acetyl-CoA-carboxylase-alpha gene induces growth inhibition and apoptosis of prostate cancer cells. Cancer Res.

[47] Swinnen JV, Beckers A, Brusselmans K, Organe S, Segers J, Timmermans L, Vanderhoydonc F, Deboel L, Derua R, Waelkens E, De Schrijver E, Van De Sande T, Noel A (2005). Mimicry of a cellular low energy status blocks tumor cell anabolism and suppresses the malignant phenotype. Cancer Res.

[48] Mabrouk GM, Helmy IM, Thampy GK, Wakil SJ (1990). Acute hormonal control of acetyl-CoA carboxylase. J Biol Chem.

[49] Luo DX, Peng X, Xiong Y, Liao DF, Cao D, Li L (2011). Dual role of insulin-like growth factor-1 in acetyl-CoA carboxylase-alpha activity in human colon cancer cells HCT-8: downregulating its expression and phosphorylation. Mol Cell Biochem.

[50] Hinton CV, Fitzgerald LD, Thompson ME (2007). Phosphatidylinositol 3-kinase/Akt signaling enhances nuclear localization and transcriptional activity of BRCA1. Exp Cell Res.

[51] Martin SA, Ouchi T (2005). BRCA1 phosphorylation regulates caspase-3 activation in UV-induced apoptosis. Cancer Res.

[52] Nelson AC, Lyons TR, Young CD, Hansen KC, Anderson SM, Holt JT (2010). AKT regulates BRCA1 stability in response to hormone signaling. Mol Cell Endocrinol.

[53] Pasanisi P, Bruno E, Venturelli E, Manoukian S, Barile M, Peissel B, De Giacomi C, Bonanni B, Berrino J, Berrino F (2011). Serum levels of IGF-I and BRCA penetrance: a case control study in breast cancer families. Fam Cancer.

[54] Neuhausen SL, Brummel S, Ding YC, Singer CF, Pfeiler G, Lynch HT, Nathanson KL, Rebbeck TR, Garber JE, Couch F, Weitzel J, Narod SA, Ganz PA (2009). Genetic variation in insulin-like growth factor signaling genes and breast cancer risk among BRCA1 and BRCA2 carriers. Breast Cancer Res.

[55] Neuhausen SL, Brummel S, Ding YC, Steele L, Nathanson KL, Domchek S, Rebbeck TR, Singer CF, Pfeiler G, Lynch HT, Garber JE, Couch F, Weitzel JN (2011). Genetic Variation in IGF2 and HTRA1 and Breast Cancer Risk Among BRCA1 and BRCA2 Carriers. Cancer Epidemiol Biomarkers Prev.

[56] Singh R, Yadav V, Kumar S, Saini N (2015). MicroRNA-195 inhibits proliferation, invasion and metastasis in breast cancer cells by targeting FASN, HMGCR, ACACA and CYP27B1. Sci Rep.

[57] Beloribi-Djefaflia S, Vasseur S, Guillaumond F (2016). Lipid metabolic reprogramming in cancer cells. Oncogenesis.

[58] Zhang F, Du G (2012). Dysregulated lipid metabolism in cancer. World J Biol Chem.

[59] Romanelli RJ, LeBeau AP, Fulmer CG, Lazzarino DA, Hochberg A, Wood TL (2007). Insulin-like growth factor type-I receptor internalization and recycling mediate the sustained phosphorylation of Akt. J Biol Chem.

[60] Perks CM, Vernon EG, Rosendahl AH, Tonge D, Holly JMP (2007). IGF-II and IGFBP-2 differentially regulate PTEN in human breast cancer cells. Oncogene.

[61] Dean SJR, Perks CM, Holly JMP, Bhoo-Pathy N, Looi LM, Mohammed NAT, Mun KS, Teo SH, Koobotse MO, Yip CH, Rhodes A (2014). Loss of PTEN expression is associated with IGFBP2 expression, younger age, and late stage in triple-negative breast cancer. Am J Clin Pathol.

[62] Burrows C, Holly JM, Laurence NJ, Vernon EG, Carter JV, Clark MA, McIntosh J, McCaig C, Winters ZE, Perks CM (2006). Insulin-like growth factor binding protein 3 has opposing actions on malignant and nonmalignant breast epithelial cells that are each reversible and dependent upon cholesterol-stabilized integrin receptor complexes. Endocrinology.

