# Supplementary material for: Elucidating the novel BRCA1 function as a non-genomic metabolic restraint in ER-positive breast cancer cell lines
Source: Oncotarget. 2018 Sep 11;9(71):33562–76. doi: 10.18632/oncotarget.26093 (PMC6173354; doi:10.18632/oncotarget.26093)
Supplement: Supplementary file 1 [file oncotarget-09-33562-s001.pdf]

Elucidating the novel BRCA1 function as a non-genomic metabolic restraint in ER-positive breast cancer cell lines

SUPPLEMENTARY MATERIALS

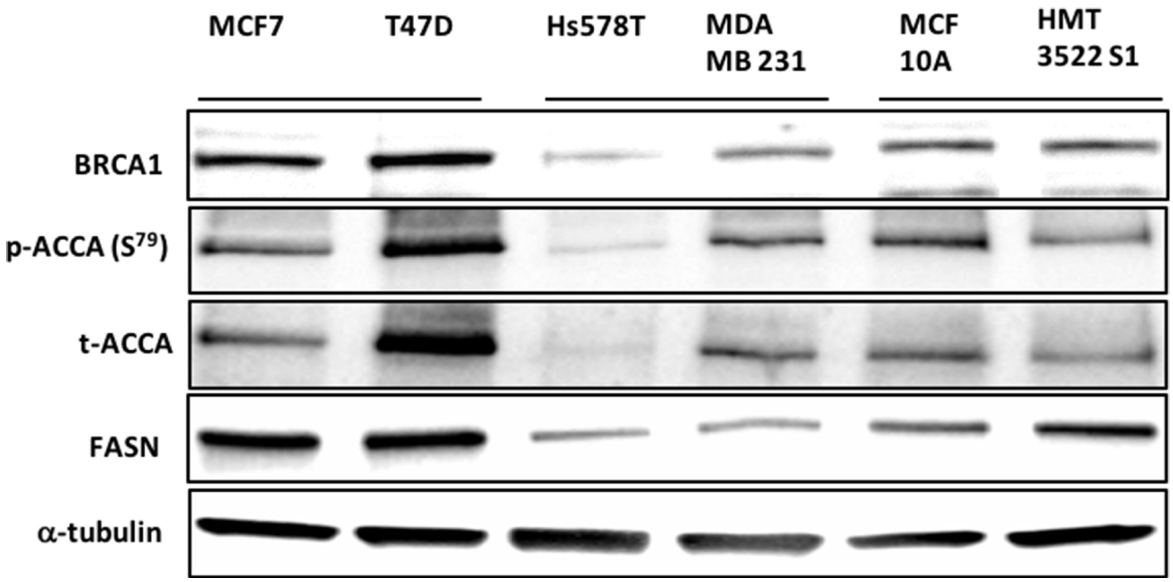

Supplementary Figure 1: The abundance of BRCA1, p-ACCA (S^{79}), ACCA and FASN proteins in breast cancer cell lines and normal mammary epithelial cells. The abundance of BRCA1, p-ACCA (S^{79}), total ACCA and FASN were analyzed using western blotting with α-tubulin used as a loading control.

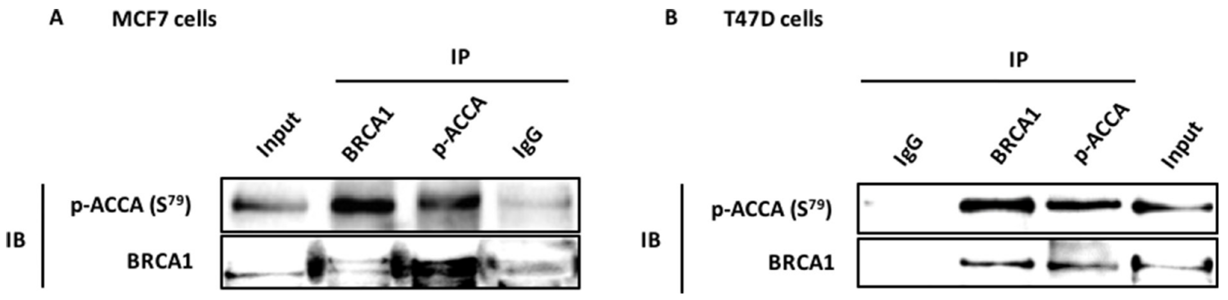

Supplementary Figure 2: BRCA1 physically associates with p-ACCA (S^{79}) in ER-positive breast cancer cell lines. (**A**) MCF7 and (**B**) T47D lysates were subjected to immunoprecipitation with either BRCA1 or p-ACCA (S^{79}) antibodies as well as control IgG. The resulting immunecomplexes were analysed by western blotting using antibodies against p-ACCA (S^{79}) and BRCA1. Representative blots from three independent experiments are shown.

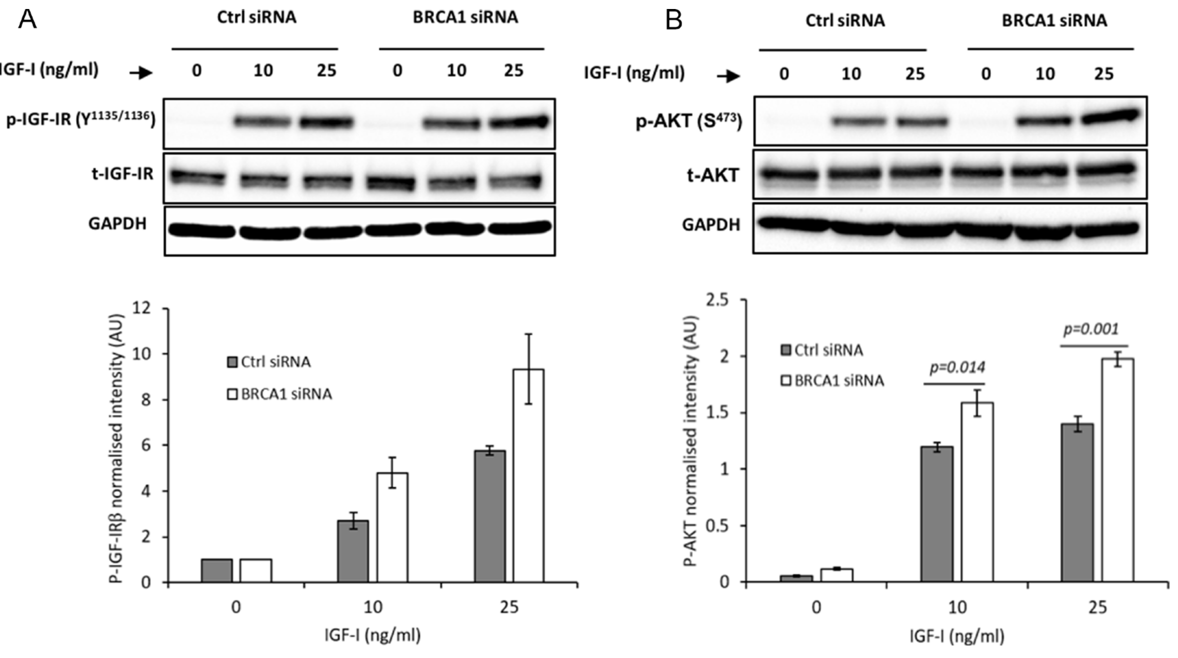

Supplementary Figure 3: BRCA1 silencing enhances IGF-I signaling in MCF7 breast cancer cells. (**A**, **B**) MCF7 breast cancer cells were transfected with BRCA1 siRNA or control siRNA for 48 hours before stimulation with 25ng/ml IGF-I for 30 minutes. Protein extracts were resolved with SDS-PAGE and immunoblotted with antibodies against (A) p-IGF-IR ($Y^{1125/1136}$) and total IGF-IR as well as (B) p-AKT (S^{473}) and total AKT. The levels of each protein were quantified using densitometry and calculated as the signal of phosphorylated protein relative to the total protein loading control. The graphs are shown below the blots and each bar represents mean ± S.E.M. of three independent experiments.
